# Wind-induced vibration serviceability of ten multi-storey timber buildings

**DOI:** 10.12688/openreseurope.21570.1

**Published:** 2025-11-06

**Authors:** Blaž Kurent, Boštjan Brank

**Affiliations:** 1 University of Ljubljana, Faculty of Civil and Geodetic Engineering, Ljubljana, Slovenia

**Keywords:** timber buildings, wind loading, building vibration, building oscillation, serviceability limit state, parametric analysis

## Abstract

Multi-storey timber buildings are prone to wind-induced vibrations, especially due to their lightweight nature, which can lead to occupant discomfort. Vibration serviceability may be a critical design criterion for such structures. In this study, the occupant comfort due to peak accelerations of wind-induced vibrations is estimated for ten European timber and hybrid timber buildings, ranging in height from 22 m to 74 m, using data on their dimensions, mass, and modal properties. The peak accelerations were computed in accordance with EN 1991-1-4:2005 and checked against ISO 10137 comfort criterion. Parametric analysis revealed that the location of the building significantly influences the expected wind-induced peak accelerations. Among the shape parameters, the radius of the rounded edges had the most substantial impact. It was found that increasing the stiffness of the building is more effective for enhancing comfort in shorter buildings, whereas adding mass is more beneficial for taller buildings. The analysis also included the current world’s tallest timber building, demonstrating the potential for constructing even taller timber structures with good occupant comfort in strong winds.

## 1. Introduction

The structural design of multi-storey buildings is often governed by the serviceability limit state criteria ^1^. Understanding how to meet these criteria efficiently increases occupant comfort and contributes to reduced material consumption and more cost-effective design decisions. Several aspects are included in the consideration of serviceability, such as vertical deflection, lateral drift of the walls, floor vibration, and vibration of the whole building ^2^. The latter may cause a separation or cracking of the exterior cladding, doors, windows, and damage to interior components, or simply induce discomfort. In timber buildings, wind-induced vibration is a common problem ^3– 5^.

Comfort criteria for assessing serviceability are based on human perception of vibration ^6^, which varies considerably between individuals. The level of vibration can be characterised by different motion properties (such as displacement, velocity, acceleration, and higher derivatives of displacement) ^7^, but accelerations are commonly used as a measure of comfort. In this context, two ISO standards are available for assessing the acceptability of vibration - ISO 10137 ^8^ and ISO 6897 ^9^. Both provide a frequency-dependent limit curve of the acceptable vibration, however, ISO 6897 only considers frequencies up to 1 Hz whereas ISO 10137 limit curve extends to 5 Hz.

The calculation of wind-induced accelerations according to EN 1991-1-4:2005 (hereafter EC1) ^10^ requires, among other assumptions, the determination of the natural frequencies of the building. For buildings higher than 50 meters, EC1 proposes a simplified empirical equation for calculating the fundamental natural frequency (in hertz) by

f=46/h,(1)

where *h* is the height of the building (in meters). The equation was derived in a study by Ellis ^11^ on the basis of 163 buildings, which did not include modern timber buildings. Larsson *et al*. ^12^ have recently evaluated the equation on 25 timber and hybrid timber buildings and found that it generally underestimates the natural frequency of a building. They also proposed a new empirical equation for the purpose of vibration serviceability check, where

f=60/h(2)

is the most probable natural frequency of a timber or hybrid timber building, and

f=50/h(3)

is a conservative estimation.

For a more accurate calculation of the modal properties, finite element (FE) modelling is necessary ^13^, using shell elements in the case of cross-laminated timber (CLT) and beam elements in the case of glued-laminated beams (GLT). However, even with detailed FE modelling, there are many sources of uncertainty when it comes to timber buildings. The first are material properties of timber. The high variability between individual (nominally identical) elements is mainly due to the inhomogeneous structure consisting of cellulose, hemicellulose and lignin, which vary considerably in stiffness and density. Depending on their contents in the wood, the material properties vary. For instance, the density of Norway spruce at 12 % moisture content typically falls between 350 and 600 kg */*m ^3^, while the along-the-grain elastic modulus varies between 13.5 and 16.7 GPa ^14^. When considering engineered wood products like CLT panels, the uncertainty of bonds between timber boards is introduced. The in-plane shear modulus varies widely among different manufacturers given by their European technical assessments (ETA), ranging from 250 MPa to 500 MPa ^15– 17^. On the other hand, mean values of 650 MPa and 450 MPa are recommended ^18^ depending on whether the narrow sides of the lamellae are glued together or not. Furthermore, wood is a highly anisotropic material due to its fibrous structure. This results in significantly higher along-the-grain stiffness than perpendicular-to-the-grain stiffness, for example, in Norway spruce, the factor between them is between 20 and 30 ^14^. In case of platform frame type buildings, the perpendicular-to-the-grain stiffness of floor slabs significantly influences the overall stiffness of the building ^19^.

In addition to the material properties of the timber, the stiffness of the connections between the timber elements must also be considered. In an earthquake, these connections have a major influence on the dynamic behaviour of the building, as they dissipate energy ^20^. Many monotonic and cyclic tests have been carried out to understand the behaviour of steel connections under extreme dynamic loads ^21– 25^. It was found that rocking is a leading failure mechanism in monotonic tests, especially for CLT panels with a small width-to-height ratio ^26^. However, when vertical loading is considered, the rocking of CLT panels does not occur until a certain threshold lateral load is exceeded ^27^. Whether the wind-induced vibrations exceed the level of activating the rocking mechanism is not yet known. In a long-term monitoring study of a timber building that recorded an earthquake ^28^, it was observed that the natural frequency was 15 % lower under seismic excitation than under wind loading. Whether the difference is due to rocking of the CLT panels and non-linear behaviour of connections is not known. Attempts at identifying the influence of connections in small amplitude vibration of timber buildings have been made ^13, 29, 30^, however, the findings are contrasting.

Even with an accurate FE model, it should be emphasised that the natural frequencies of timber buildings are subject to a significant seasonal variation. In a three-year monitoring of a four-storey hybrid timber-concrete building ^31^, a 10 % variation in the fundamental natural frequency was observed. Another long-term monitoring study of an eight-storey CLT building ^32^ found around 6 % variation in the fundamental natural frequency. Both studies observed a positive correlation between the natural frequency and the moisture content. This is also in line with the findings of a study ^33^, where drying of a CLT panel caused cracks and, thus, reduced the stiffness of the panel. Swelling of the wood due to increased moisture content resulted in higher stiffness.

Besides natural frequency, the calculation of peak accelerations according to EC1 also requires an assumption about damping. EC1 already proposes values of logarithmic decrement for reinforced concrete and steel buildings, but it does not have a recommendation for timber buildings. The design engineers of timber buildings are left to take the value provided for timber bridges or explore the scientific literature. The latter, however, records a large interval of measured values, ranging from 0.5 % to 4.0 % of critical damping ^32, 34, 35^. Some efforts have also been made to obtain a global damping value using a detailed FE modelling approach ^36^.

This paper focuses on the wind-induced vibration serviceability of timber buildings. Data of ten existing European timber and hybrid timber building designs are used, including actual measured natural frequencies and damping ratios. The aim of the paper is to determine the sensitivity of peak accelerations to the parameters of the building (such as dimensions, radius of rounded corners, mass, and modal properties) and location (such as basic value of the wind velocity and terrain category). Furthermore, vibration serviceability of a hypothetical 100 m tall timber building is examined. Several design modifications are considered such that the building satisfies comfort criteria in both, low and high-wind velocity locations.

The structure of the paper is as follows. Section 2 presents the methodology used to compute peak accelerations according to EC1 and evaluate them against ISO 10137. Section 3 shows the results of the serviceability check of ten timber and hybrid timber buildings including the sensitivity study of important parameters. Furthermore, serviceability of a hypothetical 100m tall timber building is investigated. Finally, the findings of the paper are concluded in Section 4.

## 2. Methods

This section outlines the methodology used to evaluate the comfort criterion based on ISO 10137 and the response to wind loads according to EC1 ^10^ for ten European timber buildings. The necessary data, including dimensions, masses, fundamental natural frequencies, and damping ratios, are sourced from existing literature and summarised in Table 1.

**Table 1.** The set of ten analysed buildings. For each, the first two bending modes were included.

#	Name	Stories	*h* [m]	*M* [t]	*f* [Hz]	*d* [m]	*b* [m]	*ζ* [%]	Ref
1	Yoker	7	22	1270	2.85	31	28	1.38	19, 37
					2.93	28	31	1.74	
2	Palisaden	8	27	1000	1.88	15	23	1.50	32, 38
					2.42	23	15	1.70	
3	Moholt	9	28	1150	1.98	27	23	1.44	30, 39
					2.27	23	27	1.57	
4	Haut-Bois	8	28	900	1.88	16	21	1.76	40
					2.46	21	16	2.02	
5	Panorama 1	10	30	2400	2.15	14	26	1.37	30, 41, 42
					2.45	26	14	1.57	
6	TreedIt	12	36	7390	1.39	18.5	47	1.82	43– 45
					1.49	47	18.5	1.55	
7	Panorama 2	13	39	2640	1.30	14	26	1.44	30, 41, 42
					1.63	26	14	1.18	
8	Treet	14	49	3200	0.97	23	21	1.84	46, 47
					1.12	21	23	1.61	
9	Hyperion	16	56	7000	0.95	18	31	1.64	44, 48
					1.88	31	18	1.72	
10	Mjøstårnet	18	74	6500	0.50	17	38	1.10	29, 34
					0.54	38	17	1.50	

**Figure 1.**
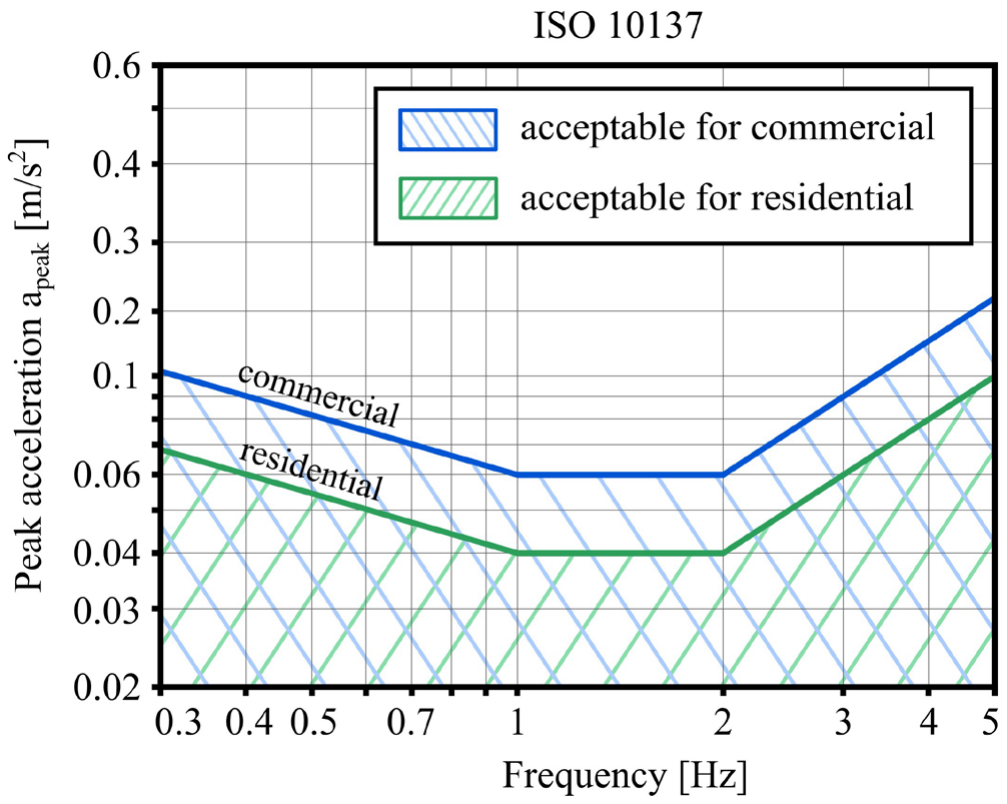
ISO 10137 comfort criteria for residential and commercial buildings.

ISO 10137 uses peak accelerations due to winds with a return period of one year to determine the acceptability of vibration. This standard distinguishes between two limit curves - one for residential buildings and one for commercial buildings. Acceptable accelerations are below the corresponding curve, as shown in Figure 1.

The peak along-wind accelerations, *a*_peak_ are calculated based on Appendix B of EC1 as

$${\text{a}}_{\text{peak}}={\text{k}}_{\text{p}}\;\sigma_{\text{a}},\;(4)$$

where *k _p_* is the peak factor (typically between 3 and 4), and *σ _a_* is the standard deviation of the along-wind accelerations. According to EC1, *σ _a_* is calculated as

$$\sigma_{a}(z)=\frac{c_{f}\rho bI_{v}(z_{s})v_{m}^{2}(z_{s})}{m_{e}}RK_{x}\;\phi (z).\;(5)$$

**Figure 2.**
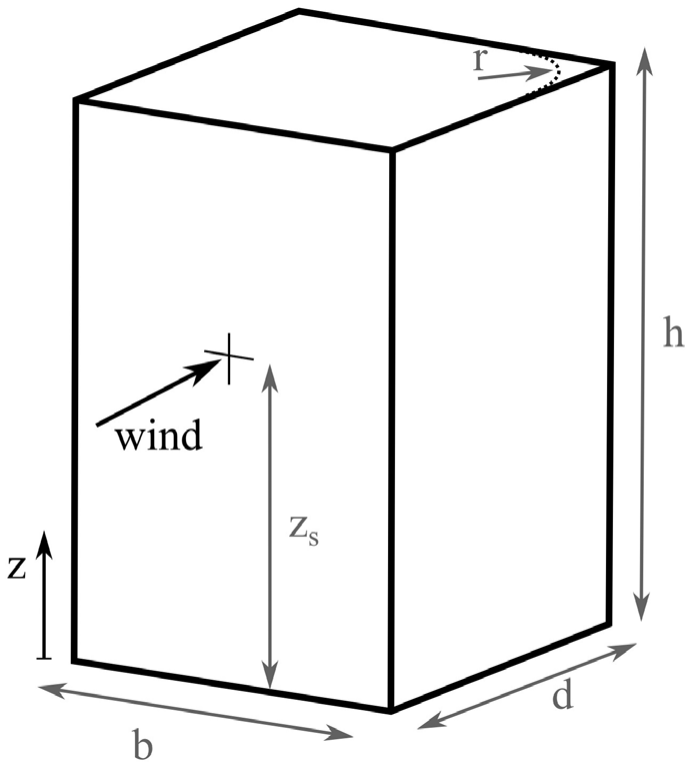
Dimensions of the building.

Here, *ρ* is the air density, *b* is the width of the building (as defined in Section 2.1), and *m _e_* is the equivalent mass (as defined in Section 2.3). The force coefficient *c _f_* depends on the shape of the building, including dimensions and radius of rounded corners. The turbulence intensity *I _v_* ( *z*) and mean wind velocity *v _m_* ( *z*) are influenced by the surrounding terrain. The latter is derived from the fundamental value of the basic wind velocity *v _b,_*_0 _which is provided by the National Annex. The modal properties of the building are considered in the resonance response *R*, the dimensionless coefficient *K _x_*, and the eigenvector *ϕ*( *z*). The *z* coordinate is defined along the building height, as shown in Figure 2, with the reference height *z _s_* for buildings defined as *z _s_* = 0 *.*6 *h*.

Parameters for computing peak accelerations can be categorised into ambient and building parameters. Ambient parameters include the fundamental value of the basic wind velocity *v _b,_*_0 _and the presence of high obstacles around the building, accounted for by selecting the terrain category (from 0 to 4 based on the height of surrounding buildings). Although terrain orography affects *v _m_* ( *z*), this paper assumes flat terrain due to the focus of the paper on the building design. Building parameters, including dimensions, modal properties, and mass, are further described in Sections 2.1 to 2.3.

### 2.1. Dimensions

For buildings with a shape of a prism, and especially those with a rectangular floor plan, the determination of the dimensions for the EC1 calculation is straightforward. The height *h* is defined as the distance to the top floor slab. In the case of Mjøstårnet, where there is an additional truss structure on the roof, this has been ignored. The width *b* and depth *d* of the building are defined according to the wind direction. The width is the dimension perpendicular to the wind direction, and the depth is parallel to it (as shown in Figure 2). In the case of rounded corners, the radius *r* is also taken into account (in all buildings, the assumption of sharp corners is the default). For buildings which do not have a rectangular floor plan (buildings 1, 3, and 6), the dimensions of the smallest rectangle that encloses the shape of the actual floor plan are used for the width and depth.

### 2.2. Modal properties

To compute the dynamic response of a building, modal properties such as the first bending natural frequency, the associated mode shape, and damping are required. While EC1 provides a simplified calculation of the natural frequency of the building using Equation (1), this analysis uses the measured natural frequencies of the buildings, as shown in Table 1.

The mode shape of the building can be represented as a normalized displacement *ϕ* along the building

*z* coordinate, given by

$$\phi (z)={\left(\frac{z}{h}\right)}^{\xi },\;(6)$$

where *ξ* is the exponent defining the shape of the eigenvector. EC1 recommends values of *ξ* between 0.6 and 1.5 for different building types.

Damping is a crucial determinant of the resonant behaviour. EC1 considers damping in the form of a logarithmic decrement *δ*, defined as the sum of

$$\delta =\delta_{s}+\delta_{a}+\delta_{d},\;(7)$$

where *δ _s_* is the logarithmic decrement of structural damping, *δ _a_* is the logarithmic decrement of aerodynamic damping, and *δ _d_* is the logarithmic decrement of damping due to special devices (such as tuned mass dampers). EC1 suggests different values of *δ _s_* depending on the type and material of the structure, but does not provide specific recommendations for timber buildings. Therefore, the damping is determined from the measured viscous damping ratio *ζ* ^49^ by

$$\delta =\frac{2\pi \zeta }{\sqrt{1-}\zeta^{2}}.\;(8)$$

The damping ratios *ζ* of the analysed buildings are shown in Table 1.

### 2.3. Equivalent mass

The equivalent mass *m _e_*, required in Equation (5), is defined in EC1 as

$$m_{e}=\frac{\int_{0}^{h}m(z)\phi^{2}(z)\;dz}{\int_{0}^{h}\phi^{2}(z)\;dz}.\;(9)$$

When the mass per unit length *m*( *z*) is evenly distributed, the calculation simplifies to $m_{e}=\frac{M}{h}$ , where *M* is the total mass of the building. For unevenly distributed mass, EC1 proposes a simplification where *m _e_* is taken to be the mass per unit length in the upper third of the building.

**Figure 3.**
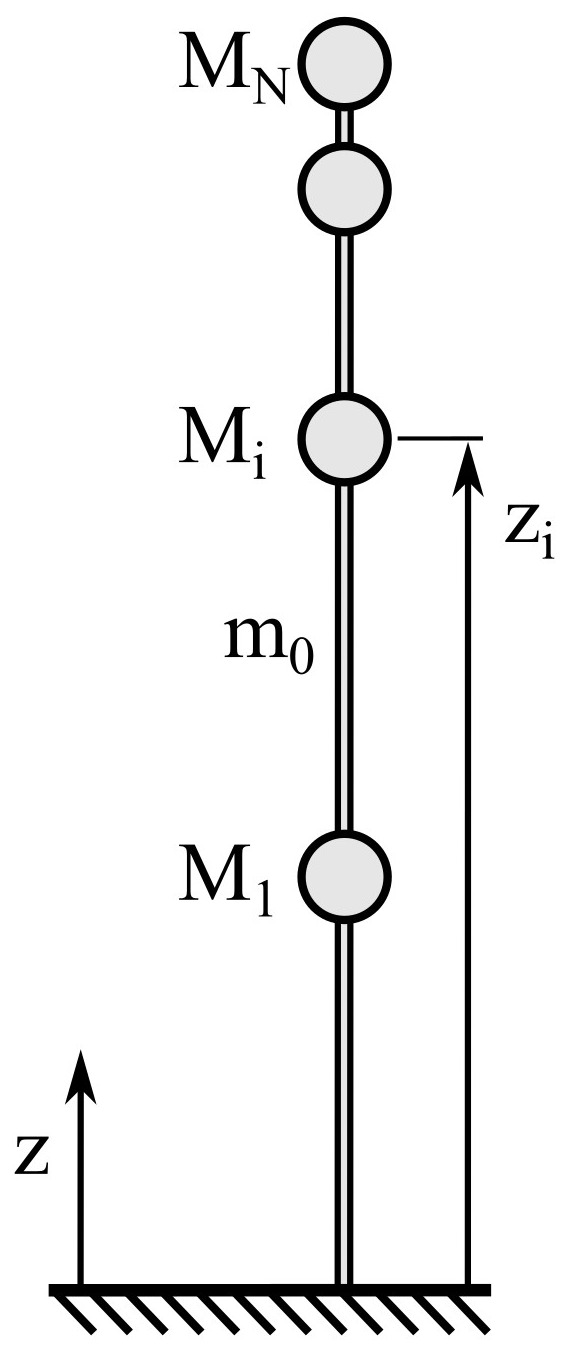
Combination of evenly distributed mass *m*_0_ and N discrete masses *M _i _* at heights *z _i_*.

For a more accurate calculation in the case of unevenly distributed mass, the assumption of a combination between the evenly distributed mass to $m_{e}=\frac{M}{h}$ and *N* discrete masses *M _i_* located at heights *z _i_* can be used, as shown in Figure 3. The mass per unit length is defined as

$$m(z)=m_{0}+\sum_{i=1}^{N}M_{i}\delta_{\text{Dirac}}(z-z_{i}),\;(10)$$

where *δ*_Dirac_( *z*) is the Dirac delta function. Using this definition, Equation (9) translates to

$$m_{e}=\frac{\int_{0}^{h}m_{0}\;\phi^{2}(z)\;dz+\int_{0}^{h}\sum_{i=1}^{N}M_{i}\delta_{\text{Dirac}}(z-z_{i})\phi^{2}(z)\;dz}{\int_{0}^{h}\phi^{2}(z)\;dz}.\;(11)$$

Subject to the rules of integration of the sum and product by a constant, Equation (11) becomes

$$m_{e}=m_{0}+\sum_{i=1}^{N}\frac{M_{i}\int_{0}^{h}\delta_{\text{Dirac}}(z-z_{i})\phi^{2}(z)\;dz}{\int_{0}^{h}\phi^{2}(z)\;dz}.\;(12)$$

Given the rule for integrating the Dirac delta function, the mass per unit length is computed as

$$m_{e}=m_{0}+\sum_{i=1}^{N}\frac{M_{i}\phi^{2}(z_{i})}{\int_{0}^{h}\phi^{2}(z)\;dz}.\;(13)$$

Assuming an eigenvector of the form given in Equation (6), the integral $\int_{0}^{h}\phi^{2}(z)$ *dz* simplifies to

$$\int_{0}^{h}\phi^{2}(z)\;dz={\int_{0}^{h}\left(\frac{z}{h}\right)}^{2\xi }\;dz=\frac{1}{h^{2\xi }}\int_{0}^{h}z^{2\xi }\;dz=\frac{1}{h^{2\xi }}{\left[\frac{z^{2\xi +1}}{2\xi +1}\right]}_{0}^{h}=\frac{h}{2\xi +1}.\;(14)$$

Thus, Equation (13) translates to

$$m_{e}=m_{0}+\;\sum_{i=1}^{N}\frac{M_{i}}{h}(2\xi +1){\left(\frac{z_{i}}{h}\right)}^{2\xi }.\;(15)$$

In a simple case where *ξ* = 1 (as recommended by EC1 for some buildings), and adding one point mass *M*_1 _on top of the building, equivalent mass is obtained by

$$m_{e}=m_{0}+\;3\frac{M_{1}}{h}.\;(16)$$

This equation shows that adding a mass on top of the building has a threefold effect on the equivalent mass.

## 3. Results

This section presents the results of the serviceability analysis of ten timber and hybrid timber buildings. The expected peak accelerations were calculated following Equation (4) and assessed by ISO 10137 comfort criteria. The first two modes of vibration were considered in the analysis. For the calculation, the actual dimensions, mass, and modal properties of the buildings were used, as given in Table 1. However, it needs to be emphasised that the actual wind conditions (such as the fundamental value of basic wind velocity, terrain category, and terrain orography) were not considered. The analysis focused on the comparison between building designs and their response to the same wind conditions. The comfort assessment, therefore, does not reflect the actual suitability of the analysed buildings and should be understood in the context of the assumed loading conditions. In addition, the calculations were made at the full building height *z* = *h*, but the comfort criteria are typically assured only up to the highest occupied floor (which might be lower than the roof slab).

**Figure 4.**
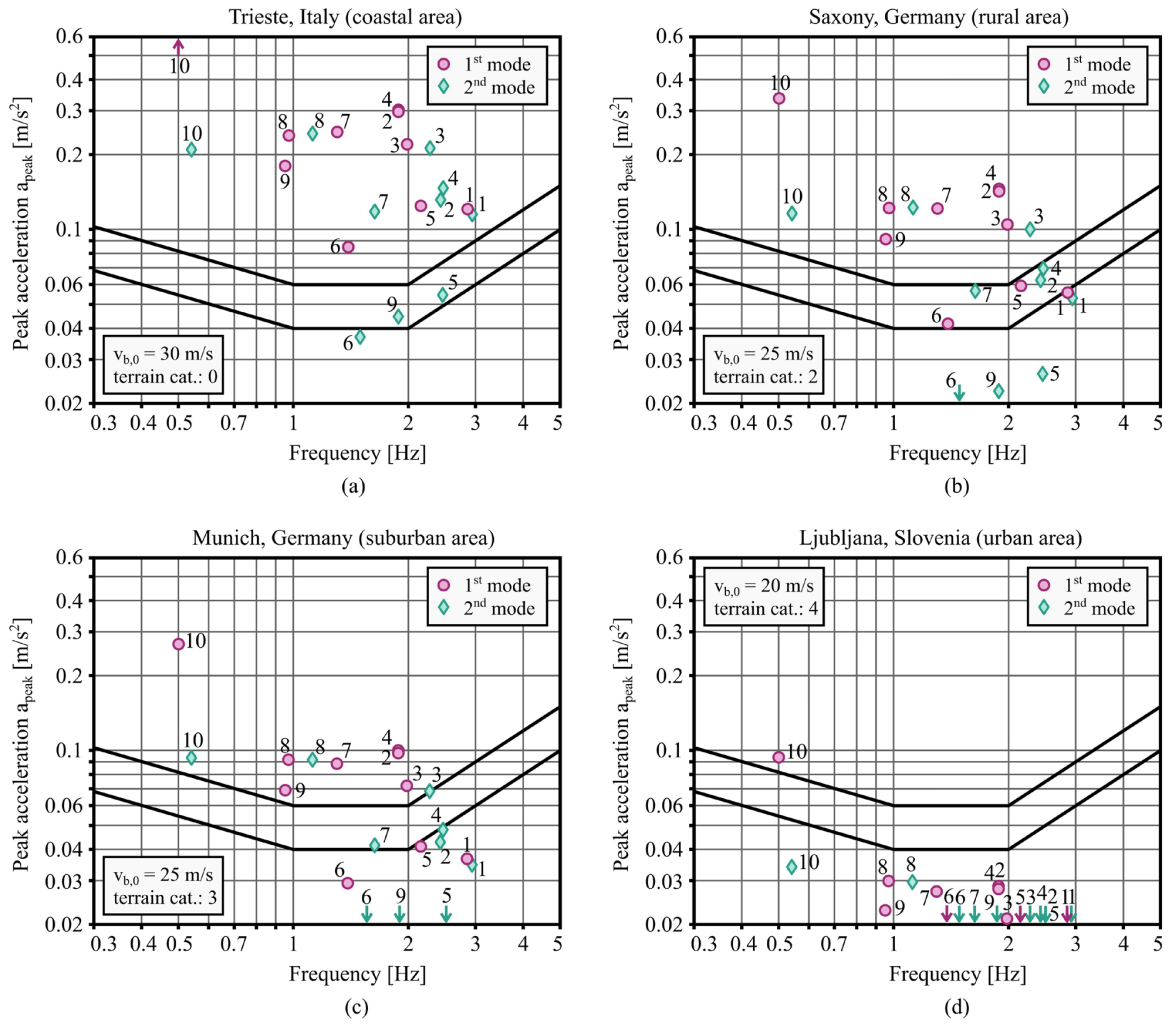
Expected peak accelerations of the ten analysed buildings depending on the wind conditions of four different locations in Europe: ( **a**) Trieste, ( **b**) Saxony, ( **c**) Munich, and ( **d**) Ljubljana.

Figure 4 shows the peak accelerations of the analysed buildings assuming different combinations of the fundamental value of the basic wind velocity *v _b,_*_0 _and the terrain category, corresponding to several European locations. The results highlight that the vibration serviceability of the buildings is highly dependent on the location. In more wind-exposed locations (such as coastal areas), where *v _b,_*_0 _= 30 m/s can be assumed, all the analysed buildings are unacceptable. On the other hand, most buildings are suitable for less windy urban areas, where *v _b,_*_0 _= 20 m/s can be assumed.

In the following sections, the results of the sensitivity analysis are presented. Section 3.1 presents the impact of terrain and wind on comfort levels, Section 3.2 shows the impact of varying dimensions of the buildings, and Section 3.3 examines the effect of modal properties. Further sensitivity analyses are carried out using *v _b,_*_0 _= 25 m/s, a terrain category of 3, and actual building properties given in Table 1 as initial parameter values.

### 3.1. Impact of the terrain

**Figure 5.**
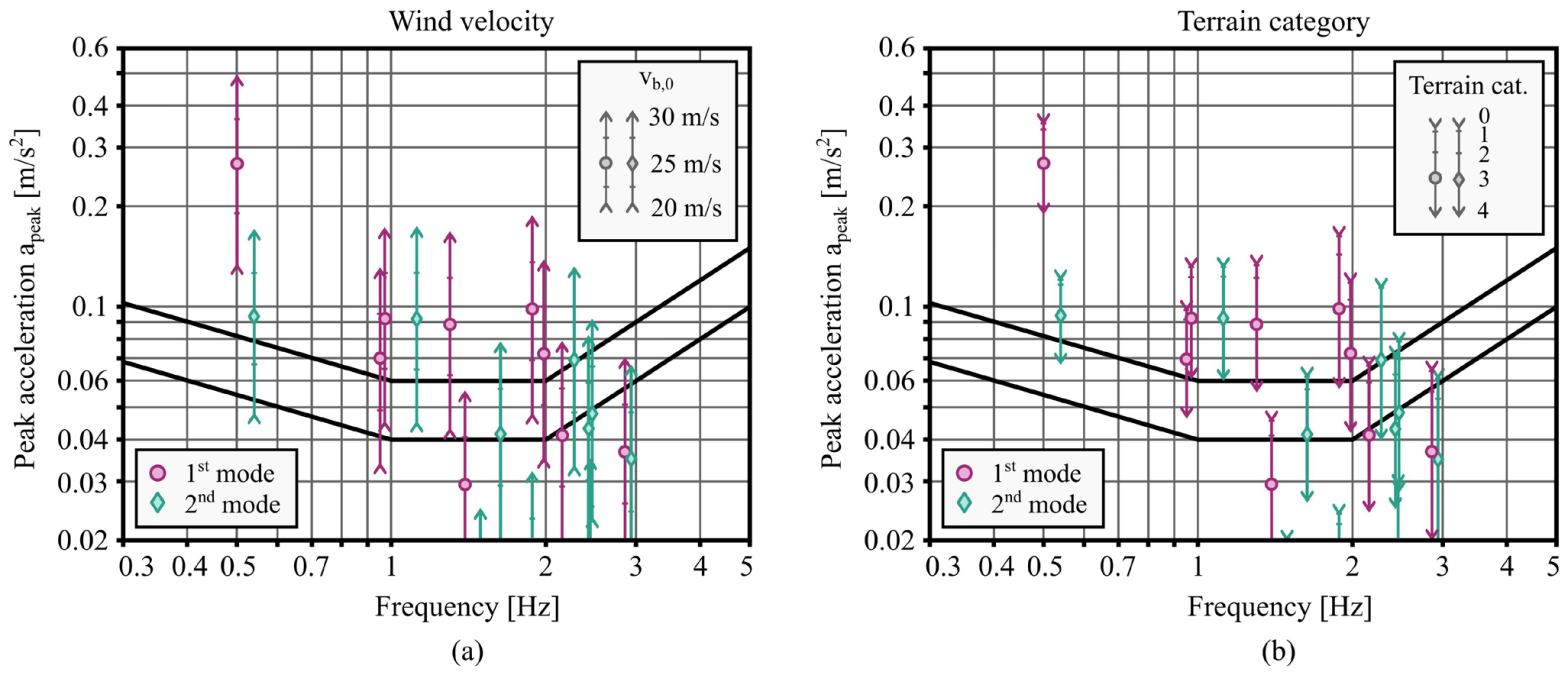
Impact of ( **a**) fundamental value of basic wind velocity *v _b,__0_* and ( **b**) terrain category on expected peak acceleration under wind loading. Plots include ISO 10137 limit curves.

The influence the location of the building has on wind-induced vibration serviceability is examined through two parameters: *v _b,_*_0 _wind velocity and terrain category. Typical values of *v _b,_*_0 _that are defined in national annexes of European countries range between 20 m */*s and 30 m */*s. In high-altitude locations, these may be significantly higher, but *v _b,_*_0 _rarely exceeds 30 m */*s in low-altitude locations. In the sensitivity analysis shown in Figure 5a *v _b,_*_0 _was varied between 20 m */*s and 30 m */*s. This resulted in around a fourfold variation in peak accelerations. In the analysis of terrain category, as shown in Figure 5b, the peak accelerations in category 0 (corresponding to exposed coastal areas) are two to three times higher compared to category 4 (corresponding to urban environments with taller obstacles). This shows that the difficulty of satisfying the comfort criteria depends to a large extent on the location of the building.

### 3.2. Impact of the shape

Given a location where wind-induced vibration serviceability will be a key governing criterion, there are multiple strategies for improving the vibration serviceability performance of the building. This section examines the impact of varying dimensions of the building. These modifications are limited by the capabilities of the site, zoning plans, as well as the expectations and resources of the investor. They should be considered in the initial stages of the building design, since later changes will be more expensive.

**Figure 6.**
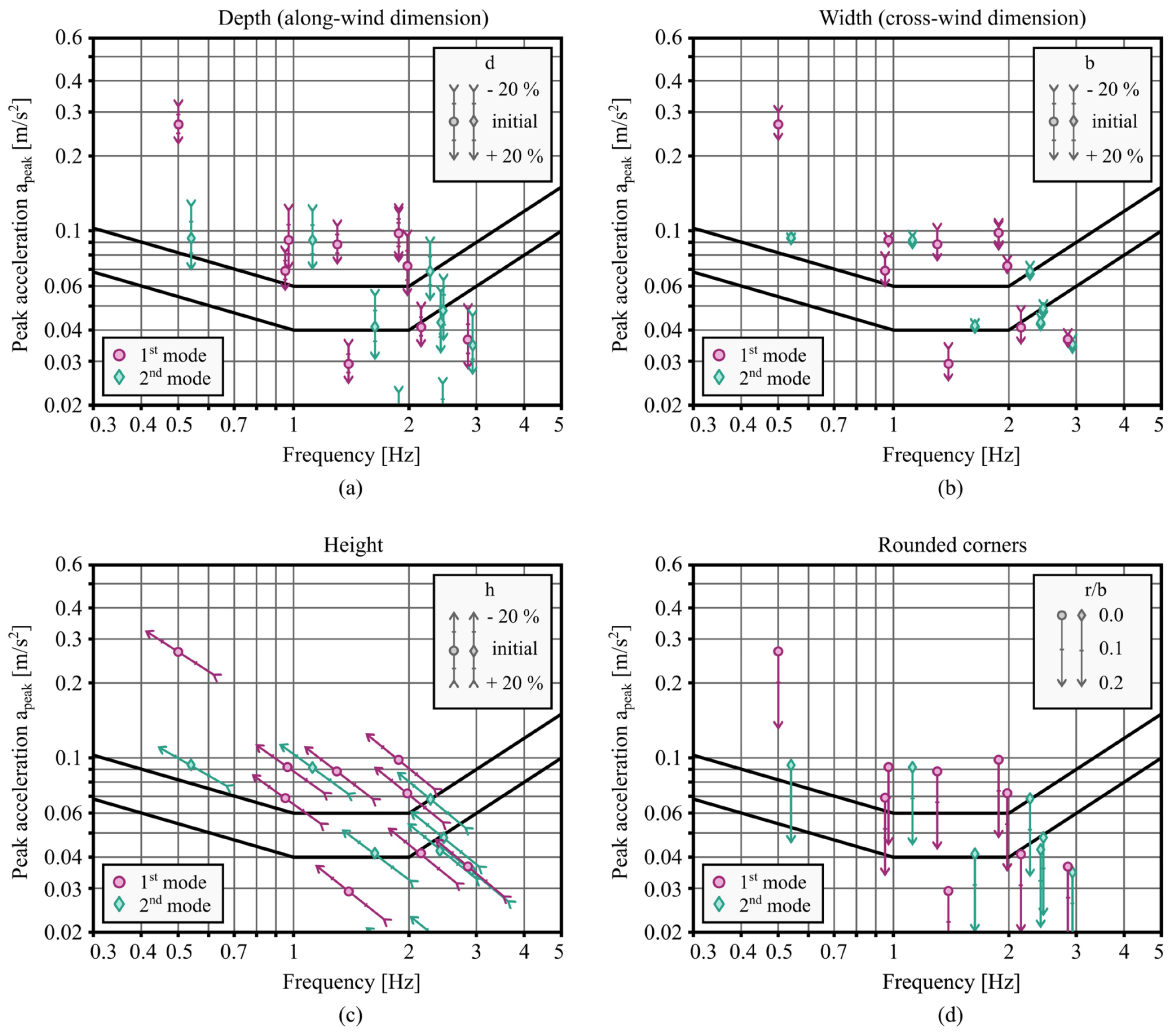
Impact of the dimensions of the building on expected peak accelerations. The parameters include ( **a**) depth, ( **b**) width, ( **c**) height, and ( **d**) radius of rounded corners. Plots include ISO 10137 limit curves.

In the sensitivity analysis, the width, depth, and height have been reduced or increased by up to 20 % from the actual dimensions. These changes were accompanied by the proportional change of the mass of the building, so that the mass per unit volume remains the same. When the height of the building was changed, the frequency was also adjusted, based on the assumption of inverse proportionality between height and natural frequency from Equation (1). I.e., when the height was increased by a factor of *x*, the natural frequency was reduced by the same factor. Conversely, it was assumed that the natural frequency remained unchanged as the width or depth of the building varied. From the results of the analyses shown in Figure 6, it can be seen that an increase in depth or width decreases the expected accelerations, while an increase in height increases them. Another interesting observation is that for buildings with natural frequencies lower than 1 Hz, the increase in the height of the building, despite the increase in accelerations, does not significantly worsen the comfort criteria due to the shape of the ISO 10137 limit curve. This suggests the possibility of building even taller timber buildings in the future.

In addition to the overall dimensions of the building, the impact of the rounded corners was analysed. The ratio of the radius of the rounded corners to the width of the building was increased up to a value of 0.2, which significantly reduced the wind force and consequently the expected accelerations of the vibration. The results are shown in Figure 6d.

### 3.3. Impact of modal properties

**Figure 7.**
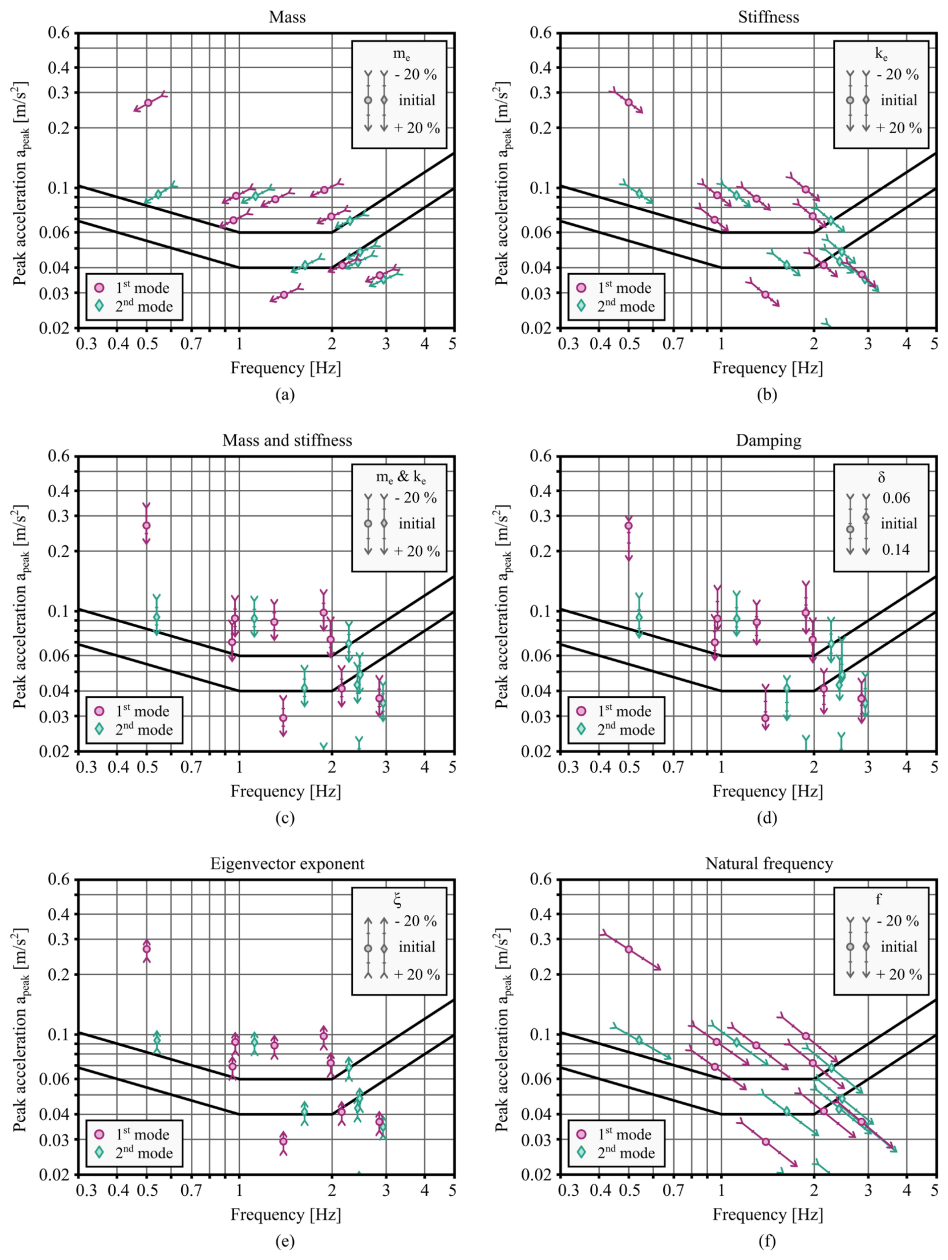
Impact of the mass, stiffness, and modal properties of the building on expected peak accelerations. The parameters include ( **a**) equivalent mass *m _e_*, ( **b**) equivalent stiffness *k _e_*, ( **c**) *m _e_* and *k _e _together*, ( **d**) logarithmic decrement *δ*, ( **e**) eigenvector exponent *ξ*, and ( **f**) natural frequency. Plots include ISO 10137 limit curves.

Finally, the influence of the modal properties is examined from two perspectives. The first is how to improve vibration serviceability by changing the mass and stiffness of the building, and the second is the perspective of the uncertainty of the prediction of modal properties. Considering the former, the natural frequency of the building can be influenced by varying the equivalent mass *m _e_* or the equivalent stiffness *k _e_*, taking into account the equation for calculating the natural frequency of a dynamic system with a single degree of freedom $f=\sqrt{\frac{k_{e}}{m_{e}}}.$ In the case of varying the mass, a constant stiffness was assumed, while in the case of varying the stiffness, a constant mass was assumed. It should be noted that the increase in equivalent mass is greater if it is added at the top of the building. Considering Equation (16), the mass added at the top of the building has three times the effect of the mass added uniformly throughout the height. Thus, a 20 % increase in equivalent mass may be achieved by adding only about 6.7 % of the total mass at the top of the building. An interesting observation in Figures 7a and 7b is that for buildings with natural frequencies below 1 Hz, adding mass is more effective, whereas for buildings with natural frequencies above 2 Hz, adding stiffness is more effective. Simultaneous proportional addition of mass and stiffness, as shown in Figure 7c, is most appropriate for buildings between 1 Hz and 2 Hz.

The modal properties of a building are extremely difficult to predict accurately at the design stage. The damping of reinforced concrete or steel buildings is determined based on the EC1 recommendation for the logarithmic decrement of structural damping. This recommendation also exists for timber bridges but not for timber buildings. From the measured data of ten buildings, the values of viscous damping *ζ* range between 1.1 % and 2.0 %, which, according to Equation (8), implies a logarithmic decrement *δ* between around 0.069 and 0.125. Even significantly higher damping values have been measured for some timber buildings ^35^, however, the parametric study considered an uncertainty of *δ* between 0.06 and 0.14. The impact of the uncertainty in the determination of damping is shown in Figure 7d. Another modal characteristic that needs to be assumed is the exponent *ξ* of the eigenvector *ϕ*( *z*). Values between 0.6 and 1.5 are recommended by EC1 for different buildings. The results of the parametric study, shown in Figure 7e, suggest this parameter is less influential in the overall calculation of peak accelerations. Finally, the effect of the natural frequency is examined. There is a considerable uncertainty associated with the prediction of the natural frequency, especially if it is obtained by the empirical approach, for example by the Equation (1). For the parametric analysis shown in Figure 7f, an error of up to 20 % has been assumed. It can be observed that the uncertainty of natural frequency has a particularly large impact on comfort levels for buildings with a frequency higher than 2 Hz. To reduce the uncertainty, more accurate approaches for predicting natural frequencies are desired.

### 3.4. Hypothetical 100 m building

Wind-induced vibration serviceability check is done for two hypothetical 100 m building designs that are based on Treet and Mjøstårnet. Each of the two designs is made in 4 variations, where different measures to reduce the peak accelerations are used. Measures used in the subsequent variations include all those from previous variations.

In variation A, the height of the building was increased up to 100 m. As a result, the frequency was adjusted based on the assumption of inverse proportionality between height and natural frequency from Equation (1). The adjusted natural frequency was thus computed as

$$f_{adjusted}=f_{real}\frac{h_{real}}{h_{adjusted}},\;(17)$$

where *f _real_* and *h _real_* are the natural frequency and height of either Treet or Mjøstårnet as defined in Table 1 and *h _adjusted_* = 100 m. The weight of the building was adjusted proportionally so that the weight per volume remained the same. Floor plan dimensions in variation A remained as defined in Table 1.

In variation B, floor plan dimensions were increased. In case of building design 1 (based on Treet) the floor plan dimensions were increased to 34 m × 34 m and in building design 2 (based on Mjøstårnet) to 38 m × 38 m. Similarly as in variation A, the weight was proportionally adjusted so that the weight per volume remained the same. The natural frequency was assumed to not be affected by the change in floor plan dimensions.

In variation C, in addition to the changes from variations A and B, equivalent mass of the building was increased by 50 %. Considering Equation (16), the mass added at the top of the building has three times the effect of the mass added uniformly throughout the height. Thus, a 50 % increase in equivalent mass may be achieved by adding about 16.7 % of the total mass at the top of the building. As shown in Section 3.3, a decrease of the natural frequency, proportional to the square root of the equivalent mass factor, can be expected. Therefore, the natural frequency was reduced by approximately 22.5 %.

Finally, corners of the building were rounded in variation D. The radius of rounded corners was set to 20 % of the width of the building. Other properties were assumed to remain unchanged from variation C.

**Figure 8.**
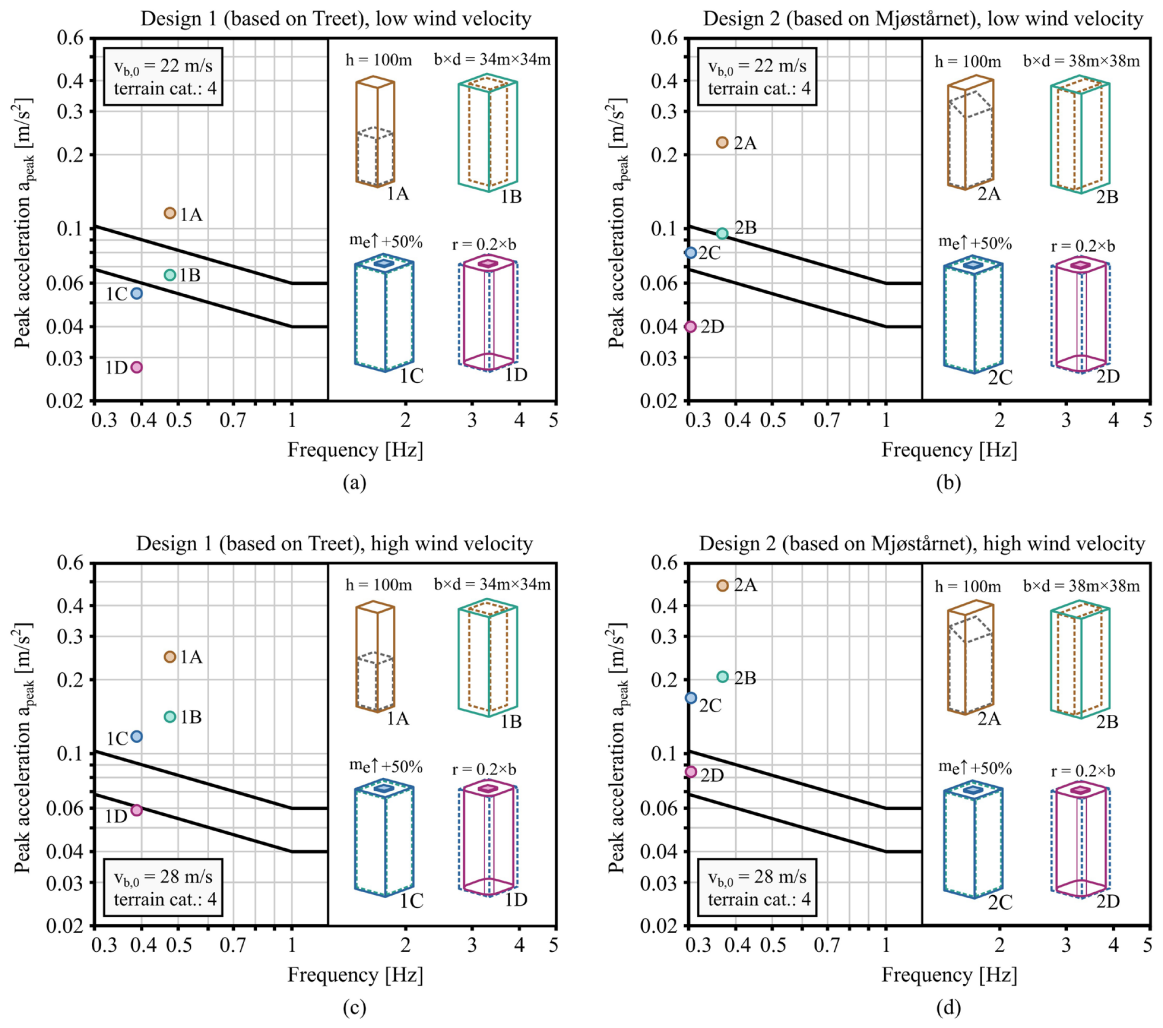
Serviceability check for a hypothetical building of 100 m with design based on Treet subfigures ( **a**) and ( **c**) - or Mjøstårnet - subfigures ( **b**) and ( **d**). Low wind velocity *v _b,_*_0_ = 22 m/s is assumed in subfigures ( **a**) and ( **b**), while high wind velocity *v _b,_*_0 _= 28 m/s in subfigures ( **c**) and ( **d**).

The building designs are checked in two wind conditions, one with *v _b,_*_0 _= 22 *m/s* and the other with *v _b,_*_0 _= 28 *m/s*, characterising low and high wind loading conditions, respectively. In both cases, terrain category 4 for urban environment with high neighbouring buildings is assumed. The results of the serviceability check are shown in Figure 8, where peak accelerations are computed for the top floor which is assumed at 95 m above ground (5 m below the architectural height of the building). It can be observed that both designs were able to satisfy the comfort criteria for residential buildings, while the severity of the required measures differed. In case of low wind loading, variation B was sufficient in design 1 and in design 2 variation C was needed. In case of high wind loading, variation D with rounded corners was needed to satisfy the comfort criteria in both designs. The results suggest that from the perspective of wind-induced vibration serviceability, building taller is possible. It should be noted, however, that other structural requirements were not considered in the analysis.

## 4. Conclusions

Based on data from ten timber and hybrid timber buildings, the expected wind-induced vibration accelerations were calculated according to EC1. The peak accelerations were used to determine the ISO 10137 comfort criterion. It was found that the location (through the expected wind velocity) has a significant influence on the difficulty of providing wind-induced vibration comfort. Most of the analysed buildings would meet the comfort criteria if they were located in a less windy urban environment (such as Ljubljana, Slovenia), but none of them would if they were located in the wind-exposed coastal area (such as Trieste, Italy). Furthermore, the parametric analysis of the building dimensions found that increasing the width or depth (with a proportional increase in mass) reduces the expected accelerations, while increasing the height of the building increases the accelerations. When analysing the modal properties, it was found that an increase in both mass and stiffness reduces the expected accelerations, but due to the frequency-dependent limit curve of the comfort criterion, stiffness has a greater effect on buildings with a first natural frequency above 2 Hz and mass on buildings below 1 Hz. Finally, a hypothetical building with a height of 100 m was analysed in multiple design variations and two wind conditions. The analysis showed that from the perspective of wind-induced vibration serviceability, building a 100 m timber building is possible. However, in high wind loading conditions, the design of a building will be heavily influenced by measures to satisfy comfort criteria.

## Ethics and consent

Ethical approval and consent were not required.

## Data Availability

All data required to reproduce the study are available within the contents of this paper. Specifically, the complete dataset, on which the analysis is based, is provided in Table 1.
