# Supplementary material for: Wind-induced vibration serviceability of ten multi-storey timber buildings
Source: Open Res Eur. 2025 Nov 6;5:337. [Version 1] doi: 10.12688/openreseurope.21570.1 (PMC12756597; doi:10.12688/openreseurope.21570.1)
Supplement: Peer review history

# Reviewer report: Reviewer response for version 1

**DOI:** 10.21956/openreseurope.23334.r66091

**Published:** 2026-01-22

**Authors:** Diego Valdivieso Cascante

**Affiliations:** 1 Pontificia Universidad Catolica de Chile, Santiago, Santiago Metropolitan Region, Chile

The paper is well written, engaging, and easy to read. The results will be of interest to the timber engineering community; however, a deeper analysis of several underlying assumptions is warranted.

 First, the study relies on a specific adopted model. It would strengthen the manuscript to discuss how alternative models available in the literature might affect the findings, particularly models developed for other contexts (e.g., the United States or Canada).

 Second, I recommend expanding the literature discussion to address friction effects, which may be further amplified by gravity load. This mechanism has been explored in timber structures and can influence both damping and the inferred response characteristics. In particular, friction can alter the effective damping, and the amplitude of the input can affect the identified damping values.

 Additionally, the introduction would benefit from a clearer discussion of standard limits and criteria for wind-induced vibration acceptability (serviceability/comfort). It may also be valuable to propagate, or at least qualitatively assess, how gravity loads and potential non-activation (or activation) of rocking mechanisms influence the predicted wind response.

 Please see the references provided in the list below:

Is the study design appropriate and does the work have academic merit?

Yes

Is the work clearly and accurately presented and does it cite the current literature?

Partly

If applicable, is the statistical analysis and its interpretation appropriate?

Partly

Are all the source data underlying the results available to ensure full reproducibility?

Yes

Are the conclusions drawn adequately supported by the results?

Yes

Are sufficient details of methods and analysis provided to allow replication by others?

Yes

Reviewer Expertise:

Timber engineering, dynamic response of timber structures

I confirm that I have read this submission and believe that I have an appropriate level of expertise to confirm that it is of an acceptable scientific standard, however I have significant reservations, as outlined above.

## References

[1] : State-of-the-Art Review on Damping in Wood-Frame Shear Wall Structures. *Journal of Structural Engineering* .2018;144(12) : 10.1061/(ASCE)ST.1943-541X.0002212 10.1061/(ASCE)ST.1943-541X.0002212

[2] : Shake table testing for system effects analysis in a 1:2 scale three-story light frame timber building. *Earthquake Spectra* .2025;41(5) : 10.1177/87552930251353813 3695-3723 10.1177/87552930251353813

[3] Labonnote, N. Damping in Timber Structures; NTNU-trykk: Trondheim, Norway, 2012.

# Reviewer report: Reviewer response for version 1

**DOI:** 10.21956/openreseurope.23334.r66090

**Published:** 2026-01-07

**Authors:** Tanmoy Konar

**Affiliations:** 1 West Bengal Police Housing and Infrastructure Development Corporation Limited, Kolkata, India

The paper focuses on the serviceability performance of timber buildings subjected to wind-induced vibrations. Ten real-world buildings are analysed under varying design wind speeds and terrain conditions to identify the influence of key parameters on the serviceability limit state. The study is further extended to examine the response of an idealized 100 m tall building, providing additional insight into serviceability behaviour at greater heights. The paper is interesting and well-written. Some minor suggestions are as follows:

 1. To better communicate with the readers, the geographical location of the example buildings, along with the corresponding design wind speeds at those locations, should be included in Table 1.

 2. A table summarizing the assumed and computed values of key parameters for the different buildings, such as m₀, M₁, Kₓ and φ, should be included to enable reproducibility and assist readers in understanding the basis of the analyses.

Is the study design appropriate and does the work have academic merit?

Yes

Is the work clearly and accurately presented and does it cite the current literature?

Yes

If applicable, is the statistical analysis and its interpretation appropriate?

Yes

Are all the source data underlying the results available to ensure full reproducibility?

Partly

Are the conclusions drawn adequately supported by the results?

Yes

Are sufficient details of methods and analysis provided to allow replication by others?

Partly

Reviewer Expertise:

Vibration control, Liquid damper

I confirm that I have read this submission and believe that I have an appropriate level of expertise to confirm that it is of an acceptable scientific standard.

# Reviewer report: Reviewer response for version 1

**DOI:** 10.21956/openreseurope.23334.r66094

**Published:** 2026-01-05

**Authors:** Abolghassem Zabihollah

**Affiliations:** 1 Tarleton State University, Stephenville, USA

This paper discusses the comfort performance of timber buildings in several European countries when subjected to wind loads. While the topic is both interesting and relevant, particularly for regions exposed to strong winds and hurricanes, it is also technically demanding. The authors are encouraged to revise the manuscript by addressing the following issues:

1. The technical terminology used throughout the manuscript is inconsistent. For example, in the results section, the term *“sensitivity analysis”* is used, whereas in the conclusion the same analysis is referred to as a *“parametric study.”* It is evident that a sensitivity analysis was not performed; therefore, the term *“parametric study”* is more appropriate and should be used consistently.
2. When presenting equations (e.g., Equation 4), the authors refer to parameter ranges specified in design standards (such as the value of *kₚ* in Equation 4), but the specific values adopted for the derivation of the results are not clearly stated.
3. Section 3.3 models the building as a single-degree-of-freedom system with equivalent mass and stiffness; however, the method used to derive the equivalent stiffness is not explained.
4. Based on the results, it appears that the building is effectively treated as a multi-degree-of-freedom system, since responses from at least two vibration modes are presented. However, the procedure used to identify and evaluate the higher modes is not adequately described. This is inconsistent with Section 3, where the structure is described as a single-degree-of-freedom system.

Is the study design appropriate and does the work have academic merit?

Yes

Is the work clearly and accurately presented and does it cite the current literature?

Yes

If applicable, is the statistical analysis and its interpretation appropriate?

Partly

Are all the source data underlying the results available to ensure full reproducibility?

Yes

Are the conclusions drawn adequately supported by the results?

Yes

Are sufficient details of methods and analysis provided to allow replication by others?

Partly

Reviewer Expertise:

Smart material and intelligent structures, structural health monitoring, vibration, and design optimization,

I confirm that I have read this submission and believe that I have an appropriate level of expertise to confirm that it is of an acceptable scientific standard.

# Reviewer report: Reviewer response for version 1

**DOI:** 10.21956/openreseurope.23334.r66095

**Published:** 2025-12-31

**Authors:** Wai Kei Ao

**Affiliations:** 1 The Hong Kong Polytechnic University, Hung Hom, Hong Kong

**Overall Comments:**

 The manuscript addresses a critical issue in the design of multi-storey timber buildings: wind-induced vibration serviceability. The novelty lies in the analysis of ten existing European timber and hybrid timber buildings, using actual measured modal data (natural frequencies and damping ratios). This empirical foundation lends significant weight to the results. The comprehensive parametric analysis, particularly the findings regarding the relative effectiveness of increasing stiffness versus adding mass based on the building's natural frequency, provides valuable technical guidance for structural designers.

 In summary, I believe this paper has some research value and practical engineering value. Therefore, the reviewer recommends a minor revision and goes for the publication process again. The reviewer will analyse the strengths and weaknesses of the paper in detail, and propose specific suggestions for revision:

**Specific Comments:**

**1. Introduction**

 a) The Introduction establishes that the natural frequencies of timber buildings are subject to significant seasonal variation (up to 10%). While the study uses measured frequencies (Table 1), it might be helpful to briefly discuss whether the reported measured frequencies account for this environmental variability, or if they represent a typical or worst-case condition, as this uncertainty is critical to the serviceability check.

 b) The authors can add in some latest vibration **serviceability** studies to enhance the literature as follows: s13349-025-00938-6, j.jobe.2025.113168, j.tws.2024.112226.

**2. Methods**

 a) The specific value or range of \xi used for the calculation of the ten buildings in the main analysis (Section 3) should be explicitly stated in this section for full reproducibility.

 b) A brief discussion or supplementary calculation comparing the EC1 methodology (used here) with other international standards would significantly enhance the academic merit and global relevance of the paper.

**3. Results**

 a) The authors must provide verification or justification (either citing literature or empirical observation) that this linear extrapolation is reasonable up to 100 m, or acknowledge the increased uncertainty introduced by this assumption.

 b) The existing reviewer suggested including analysis of the analytical vs. FE predictions of a_{peak} and investigating the conservatism of the analytical approach by checking the root-mean-square acceleration (a_{rms}). Providing a_{rms} values would offer a more comprehensive view of the vibration response.

Is the study design appropriate and does the work have academic merit?

Yes

Is the work clearly and accurately presented and does it cite the current literature?

Yes

If applicable, is the statistical analysis and its interpretation appropriate?

Yes

Are all the source data underlying the results available to ensure full reproducibility?

Yes

Are the conclusions drawn adequately supported by the results?

Yes

Are sufficient details of methods and analysis provided to allow replication by others?

Yes

Reviewer Expertise:

Structural dynamics; Structural health monitoring; Human dynamics; Smart sensing technology; Sensors and actuators; Smart transportation; Infrastructure resilience; Climate modelling and atmospheric dynamics; GeoSystems and GeoEngineering; Vibration and noise measurement; Vibration and noise control; Vibration isolation; Vibration and noise serviceability; Smart manufacture and operation; Energy harvesting; Metastructure and metamaterial; Data-driven modelling; Big data analysis; AI; Machine learning; Vision-based sensing and image processing; Finite element modelling and model updating; Digital Twin; Nonlinear dynamic and analysis; Sports science and technology; Biomechanics.

I confirm that I have read this submission and believe that I have an appropriate level of expertise to confirm that it is of an acceptable scientific standard.

# Reviewer report: Reviewer response for version 1

**DOI:** 10.21956/openreseurope.23334.r63767

**Published:** 2025-11-13

**Authors:** Angelo Aloisio

**Affiliations:** 1 Università degli Studi dell’Aquila, L’Aquila, Italy

The paper is engaging, well-written and can be considered for indexing in its current form. Some minor suggestions:

 -You only focused on Eurocode, and the performance is, in general, borderline. What about the predictive equations for a_peak from the American/Canedian standards?

 -regarding the natural freq. extrapolation up to 100m. Do you think it is worth including a verification that the linear decrease with height is linear? I know from some personal experience that in high buildings the relationship is nonlinear..

 -Do you think there is a big gap between analytical and FE predictions of the a_peak? It would be interesting to understand also the a_rms and how conservative is the analytical approach

Is the study design appropriate and does the work have academic merit?

Yes

Is the work clearly and accurately presented and does it cite the current literature?

Yes

If applicable, is the statistical analysis and its interpretation appropriate?

Yes

Are all the source data underlying the results available to ensure full reproducibility?

Yes

Are the conclusions drawn adequately supported by the results?

Yes

Are sufficient details of methods and analysis provided to allow replication by others?

Yes

Reviewer Expertise:

Structural Dynamics and Timber ENGINEERING

I confirm that I have read this submission and believe that I have an appropriate level of expertise to confirm that it is of an acceptable scientific standard.
